# Chemometric Methods for Spectroscopy-Based Pharmaceutical Analysis

**DOI:** 10.3389/fchem.2018.00576

**Published:** 2018-11-21

**Authors:** Alessandra Biancolillo, Federico Marini

**Affiliations:** Department of Chemistry, University of Rome La Sapienza, Rome, Italy

**Keywords:** spectroscopy, chemometrics and statistics, component analysis (PCA), partial least squares (PLS), classification, partial least squares discriminant analysis (PLS-DA), soft independent modeling of class analogies (SIMCA), pharmaceutical quality control

## Abstract

Spectroscopy is widely used to characterize pharmaceutical products or processes, especially due to its desirable characteristics of being rapid, cheap, non-invasive/non-destructive and applicable both off-line and in-/at-/on-line. Spectroscopic techniques produce profiles containing a high amount of information, which can profitably be exploited through the use of multivariate mathematic and statistic (chemometric) techniques. The present paper aims at providing a brief overview of the different chemometric approaches applicable in the context of spectroscopy-based pharmaceutical analysis, discussing both the unsupervised exploration of the collected data and the possibility of building predictive models for both quantitative (calibration) and qualitative (classification) responses.

## Introduction

Quality control on pharmaceutical products is undoubtedly an important and widely debated topic. Hence, in literature, various methods have been proposed to check quality of medicines, either qualitative (e.g., for the identification of an active pharmaceutical ingredient, API; Blanco et al., 2000; Herkert et al., 2001; Alvarenga et al., 2008) or quantitative (quantification of the API; Blanco et al., 2000; Yao et al., 2007; Cruz Sarraguça and Almeida Lopes, 2009); involving either destructive or non-invasive online techniques. Recently, due to the benefits they bring, several non-destructive methodologies based on spectroscopic techniques (mainly Near-Infrared NIR) combined with chemometric tools have been proposed for pharmaceutical quality check (Chen et al., 2018; Rodionova et al., 2018).

Despite the development of analytical methodologies and the commitments of national and supranational entities to regulate pharmaceutical quality control, substandard and counterfeit medicines are still a major problem all over the world.

### Chemometrics as tool for fraud/adulteration detection

Poor-quality pharmaceuticals can be found on the market for two main reasons: low production standards (mainly leading to substandard medicines) and fraud attempts. Counterfeited drugs may present different frauds/adulterations; for instance, they could contain no active pharmaceutical ingredient (API), a different API from the one declared, or a different (lower) API strength. As mentioned above, several methodologies have been proposed in order to detect substandard/counterfeit pharmaceuticals; among these, a major role is played by those based on the application of spectroscopic techniques in combination with different chemometric methods. The relevance of these methodologies is due to the fact that spectroscopy (in particular, NIR) combined with exploratory data analysis, classification and regression method can lead to effective, high performing, fast, non-destructive, and sometimes, online methods for checking the quality of pharmaceuticals and their compliance to production and/or pharmacopeia standards. Nevertheless, the available chemometric tools applicable to handle spectroscopic (but, of course not only those) data are numerous, and there is plenty of room for their misapplication (Kjeldahl and Bro, 2010). As a consequence, the aim of the present paper is to report and critically discuss some of the chemometric methods typically applied for pharmaceutical analysis, together with an essential description of the figures of merit which allow evaluating the quality of the corresponding models.

## Exploratory data analysis

**Figure 1:**
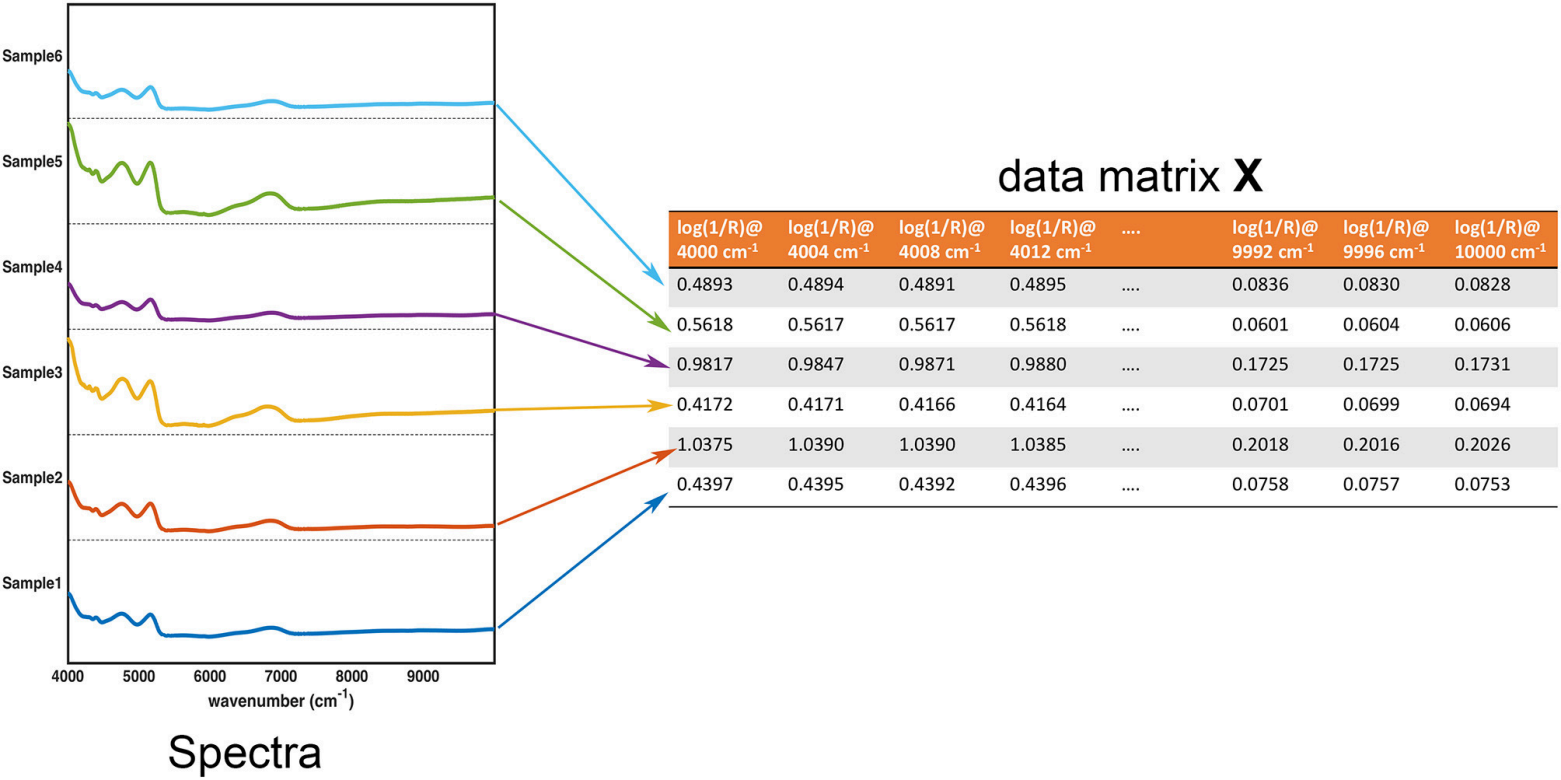
Graphical illustration of the equivalence between the collected experimental data (in this case, NIR spectra for 6 samples) and the data matrix **X**. Each row of the data matrix corresponds to the spectrum of a sample, whereas each column contains the value of a specific variable over all the individuals.

In the large part of the studies for the characterization of pharmaceutical samples for quality control, verification of compliance and identification/detection of counterfeit, fraud or adulterations, experimental signals (usually in the form of some sorts of fingerprints) are collected on a series of specimens. These constitute the data the chemometric models operate on. These data are usually arranged in the form of a matrix ***X***, having as many rows as the number of samples and as many columns as the number of measured variables. Accordingly, assuming that samples are spectroscopically characterized by collecting an absorption (or reflection/transmission) profile (e.g., in the infrared region), each row of the matrix corresponds to the whole spectrum of a particular sample, whereas each column represents the absorbance (or reflectance/transmittance) of all the individuals at a particular wavenumber. This equivalence between the experimental profiles and their matrix representation is graphically reported in Figure 1.

Once the data have been collected, exploratory data analysis represents the first step of any chemometric processing, as it allows “to summarize the main characteristics of data in an easy-to-understand form, often with visual graphs, without using a statistical model or having formulated a hypothesis” (Tukey, 1977). Exploratory data analysis provides an overall view of the system under study, allowing to catch possible similarities/dissimilarities among samples, to identify the presence of clusters or, in general, systematic trends, to discover which variables are relevant to describe the system and, on the other hand, which could be in principle discarded, and to detect possible outlying, anomalous or, at least, suspicious samples (if present). As evident also from the definition reported above, in the context of exploratory data analysis a key role is played by the possibility of capturing the main structure of the data in a series of representative plots, through appropriate display techniques. Indeed, considering a general data matrix ***X***, of dimensions *N* × *M*, one could think of its entries as the coordinates of *N* points (the samples) into a *M*-dimensional space whose axes are the variables, which makes this representation unfeasible for the cases when more than three descriptors are collected on each individual. This is why exploratory data analysis often relies on the use of projection (bilinear) techniques to reduce the data dimensionality in a “clever” way. Projection methods look for a low-dimensional representation of the data, whose axes (normally deemed components or latent variables) are as relevant as possible for the specific task. In the case of exploratory data analysis, the most commonly used technique is Principal Components Analysis (PCA) (Pearson, 1901; Wold et al., 1987; Jolliffe, 2002).

### Principal component analysis

Principal component analysis (PCA) is a projection method, which looks for directions in the multivariate space progressively providing the best fit of the data distribution, i.e., which best approximate the data in a least squares sense. This explains why PCA is the technique of choice in the majority of cases when exploratory data analysis is the task: indeed, by definition, for any desired number of dimensions (components) *F* in the final representation, the subspace identified by PCA constitutes the most faithful *F*-dimensional approximation of the original data. This allows compression of the data dimensionality at the same time reducing to a minimum the loss of information. In particular, starting from a data matrix ***X***_(*N* × *M*)_, Principal Component Analysis is based on its bilinear decomposition, which can be mathematically described by Equation (1):

(1) $$X=TP^{T}+E$$

The *loadings* matrix ***P***_(*M* × *F*)_ identifies the *F* directions, i.e., the *principal components* (PC), along which the data should be projected and the results of such projection, i.e., the coordinates of the samples onto this reduced subspace, are collected in the *scores matrix* ***T***_(*N* × *F*)_. In order to achieve data compression, usually *F*≪*M* so that the PCA representation provides an approximation of the original data whose residuals are collected in the matrix ***E***_(*N* × *M*)_.

**Figure 2:**
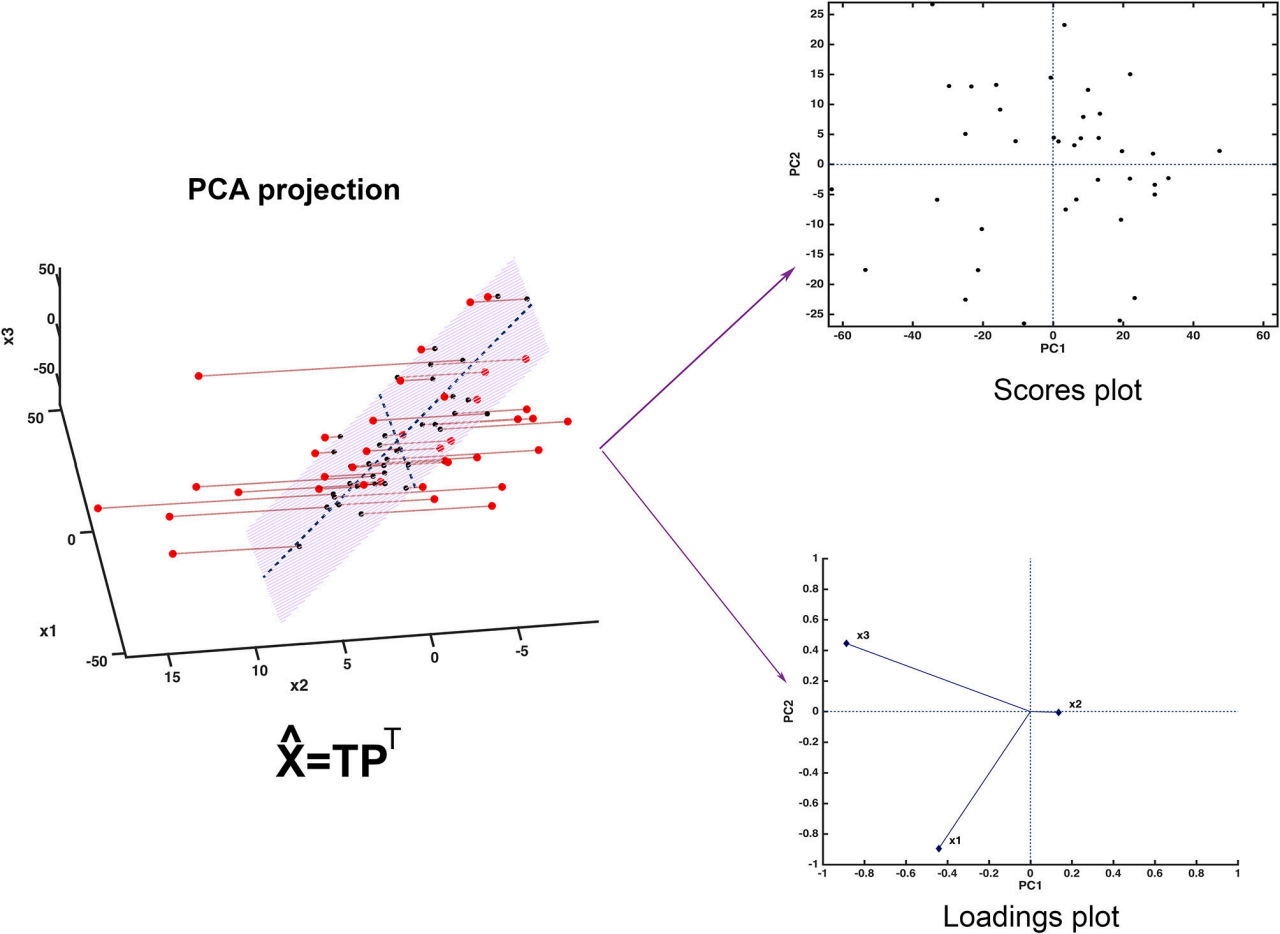
Graphical illustration of the basics of PCA. The samples, here represented in a three-dimensional space, are projected onto a low-dimensional subspace (highlighted in light red in the leftmost panel) spanned by the first two principal components. Inspection of the data set can be carried out by looking at the distribution of the samples onto the informative PC subspace (scores plot) and interpretation can be then carried out by examining the relative contribution of the experimental variable to the definition of the principal components (loadings plot).

Since the scores represent a new set of coordinates along highly informative (relevant) directions, they may be used in two- or three-dimensional scatterplots (scores plots). This offers a straightforward visualization of the data, which can highlight possible trends in data, presence of clusters or, in general, of an underlying structure. A schematic representation of how PCA works is displayed in Figure 2.

**Figure 3:**
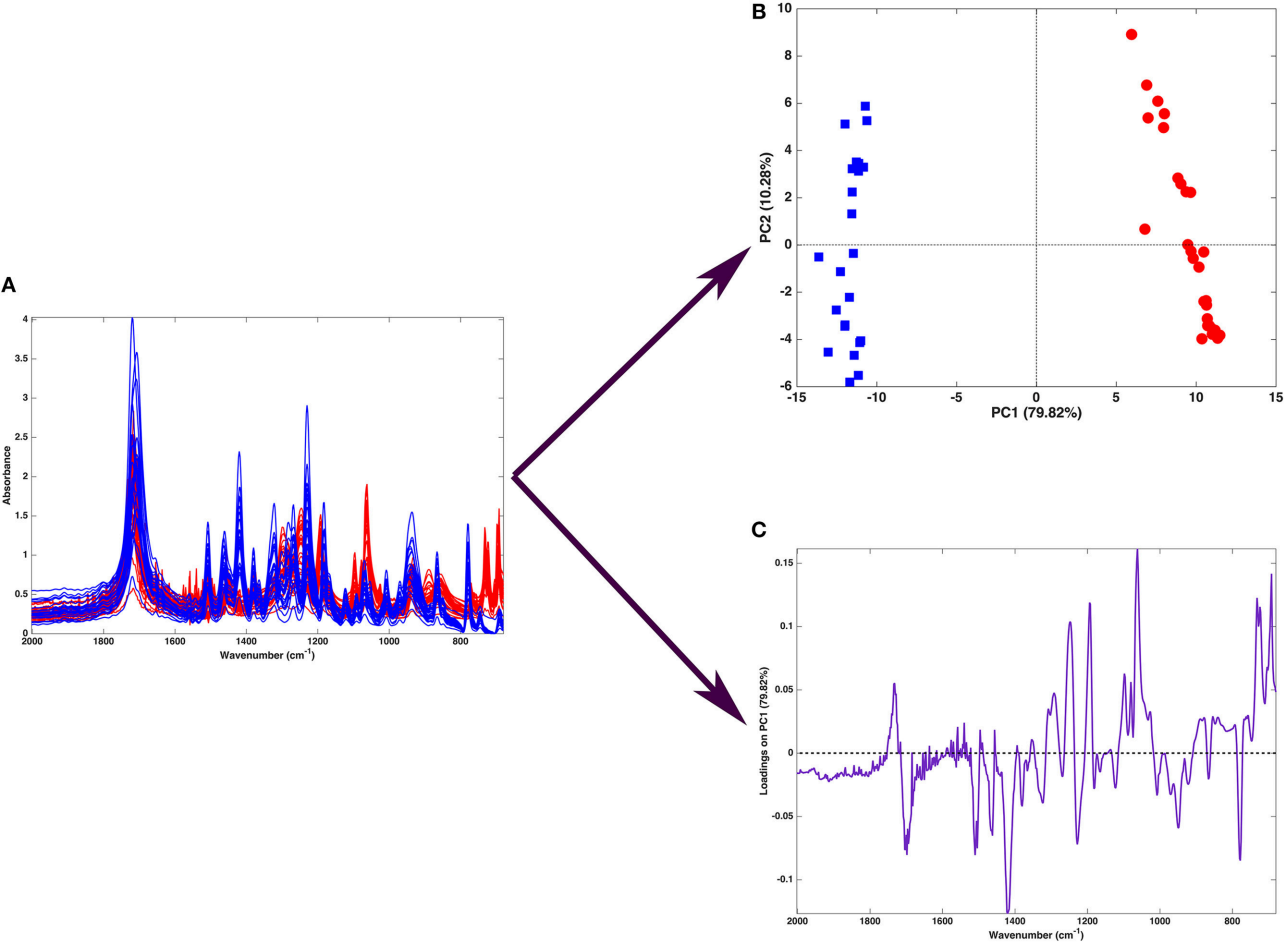
Graphical illustration of the application of PCA on a spectral (mid-infrared) data. Fifty-one spectra recorded on samples containing either ibuprofen (blue) or ketoprofen (red) are recorded in the region 680–2,000 cm^−1^ **(A)**. When PCA is applied to such a dataset, one obtains a scores plot **(B)** showing that two cluster of samples, corresponding to tablets containing ibuprofen (blue squares) or ketoprofen (red circles) are separated along the first component. Interpretation of the observed differences in terms of the spectroscopic signal is made possible by the inspection of the loadings on PC, which are shown in a “spectral-like” fashion in **(C)**.

Figure 2 shows one of the simplest possible examples of feature reduction, since it describes the case where samples described by three measured variables can be approximated by being projected on an appropriately chosen two-dimensional sub-space. However, the concept may be easily generalized to higher-dimensional problems, such as those involving spectroscopic measurements. Figure 3 shows an example of the application of PCA to mid infrared spectroscopic data. In particular, the possibility of extracting as much information as possible from the IR spectra recorded on 51 tablets containing either ketoprofen or ibuprofen in the region 2,000–680 cm^−1^ (661 variables) is represented.

A large portion of the data variability can be summarized by projecting the samples onto the space spanned by the first two principal components, which account for about 90% of the original variance, and therefore can be considered as a good approximation of the experimental matrix. Inspection of the scores plot suggests that the main source of variability is the difference between ibuprofen tablets (blue squares) and ketoprofen ones (red circles), since the two clusters are completely separated along the first principal component. To interpret the observed cluster structure in terms of the measured variables, it is then necessary to inspect the corresponding loadings, which are also displayed in Figure 3 for PC1. Indeed, for spectral data, the possibility of plotting the loadings for the individual components in a profile-like fashion, rather than producing scatterplot for pairs of latent variables (as exemplified in Figure 2) is often preferred, due to its more straightforward interpretability: spectral regions having positive loadings will have higher intensity on samples which have positive scores on the corresponding component, whereas bands associated to negative loadings will present higher intensity on the individuals falling at negative values of the PC. In the example reported in Figure 3, one could infer, for instance, that the ketoprofen samples (which fall at positive values of PC1) have a higher absorbance at the wavenumbers where the loadings are positive, whereas ibuprofen samples should present a higher signal in correspondence to the bands showing negative loadings.

Based on what reported above, it is evident how the quality of the compressed representation in the PC space depends on the number of components *F* chosen to describe the data. However, at the same time, it must be noted that when the aim of calculating a PCA is “only” data display, as in most of the applications in the context of exploratory analysis, the choice of the optimal number of components is not critical: it is normally enough to inspect the data distribution across the first few dimensions and, in many cases, considering the scores plot resulting from the first two or three components could be sufficient. On the other hand, there may be cases when the aim of the exploratory analysis is not limited to just data visualization and, for instance, one is interested in the identification of anomalous or outlying observations, or there could be the need of the imputation of missing elements in the data matrix; additionally, one could also need to obtain a compressed representation of the data to be used for further predictive modeling. In all such cases, the choice of the optimal dimensionality of the PC representation is critical for the specific purposes and, therefore, the number of PCs should be carefully estimated. In this respect, different methods have been proposed in the literature and a survey of the most commonly used can be found in Jolliffe (2002).

Among the applications described above, the possibility of using PCA for the identification/detection of potential outliers deserves a few more words, as it could be of interest for pharmaceutical quality control. Actually, although outliers—or anomalous observations, in general—could be, in principle, investigated by visually inspecting the scores plot along the first components, this approach could be subjective and anyway would not consider some possible data discrepancies. Alternatively, when it is used as a model to build a suitable approximation of the data, PCA provides a powerful toolbox for outlier detection based on the definition of more objective test statistics, which can be easily automatized or, anyway, embedded in control strategies, also on-line. This is accomplished by defining two distance measurements: (i) a squared Mahalanobis distance in the scores space, which follows the T^2^ statistics (Hotelling, 1931) and accounts for how extreme the measurement is in the principal component subspace, and (ii) a squared orthogonal Euclidean distance (the sum of squares of the residuals after approximating the observation by its projection), which is normally indicated as Q statistics (Jackson and Muldholkar, 1979) and quantifies how well the model fits that particular individual. Outlier detection is then carried out by setting appropriate threshold values for the T^2^ and Q statistics and verifying whether the samples fall below or above those critical limits. Moreover, once an observation is identified as a potential outlier, inspection of the contribution plot can help in relating the detected anomaly to the behavior of specific measured variables.

#### Selected examples

PCA is customarily used for the quality control of drugs and pharmaceuticals; several examples of the application of this technique to solve diverse issues have been reported in the literature. One of the most obviously relevant ones is fraud detection. For example, in Rodionova et al. (2005) PCA was applied to both bulk NIR spectroscopy and hyperspectral imaging (HSI) in the NIR range to spot counterfeit drugs. In particular, bulk NIR was used to differentiate genuine antispasmodic drugs from forgeries, whereas HSI on the ground uncoated tablets was employed to identify counterfeited antimicrobial drugs. In both cases, the spectroscopic data were subjected to PCA, which allowed to clearly identify clusters in the scores plot, corresponding to the two kinds of tablets, i.e., genuine and counterfeited. In the case of the imaging platform, where the signal is stored as a data hypercube [i.e., a three-way numerical array of dimension number of horizontal pixels N_x_, number of vertical pixels N_y_ and number of wavelengths N_λ_, in which each entry corresponds to the spectral intensity measured at a certain wavelength and a specific spatial position (x-y coordinates)], a preliminary unfolding step is needed. Unfolding is the procedure allowing to reorganize a higher-order array into a two-way matrix, which can be then processed with standard chemometric techniques. In the case of hyperspectral data cubes, this is carried out by stacking the spectra corresponding to the different pixels one on top of each other, in a way to obtain a matrix of dimensions (N_x_ × N_y_ and N_λ_).

Another relevant application of exploratory analysis is related to quality check. For instance, PCA can be applied to investigate formulations not meeting predefined parameters. In Roggo et al. (2005), PCA was used to inquire a suspicious blue spot present on tablets. Samples were analyzed by a multi-spectral (IR) imaging microscope and PCA analysis was performed on the unfolded data-cube, indicating that the localized coloration was not due to contamination, but actually given by wet indigo carmine dye and placebo (expected ingredients of the formulation).

PCA can also be used for routine quality checks at the end of a production process. For example, in Myakalwar et al. (2011) laser-induced breakdown spectroscopy (LIBS) and PCA were combined with the aim of obtaining qualitative information about the composition of different pharmaceuticals.

## Regression

As discussed in the previous section, exploratory analysis is a first and fundamental step in chemometric data processing and, in some cases, it could be the only approach needed to characterize the samples under investigation. However, due to its unsupervised nature, it provides only a (hopefully) unbiased picture of the data distribution but it lacks any possibility of formulating predictions on new observations, which on the other hand may be a fundamental aspect to solve specific issues. In practice, very often quality control and/or authentication of pharmaceutical products rely on some forms of qualitative or quantitative predictions. For instance, the quantification of a specific compound (e.g., an active ingredient or an excipient) contained in a formulation is a routine operation in pharmaceutical laboratories. This goal can be achieved by combining instrumental (e.g. spectroscopic) measurements with chemometric regression approaches (Martens and Naes, 1991; Martens and Geladi, 2004). Indeed, given a response to be predicted *y* and a vector of measured signals (e.g., a spectrum) ***x***, the aim of regression methods is to find the functional relationship that best approximates the response on the basis of the measurements (the *predictors*). Mathematically, this can be stated as:

(2) $$y=\hat{y}+e=f\left(x\right)+e$$

where ŷ is the predicted response (i.e., the response value approximated by the model), *f*(***x***) indicates a general function of and ***x*** and *e* is the residual, i.e., the difference between the actual response and its predicted value. In many applications, the functional relationship between the response and the predictors *f*(***x***) can be assumed to be linear:

(3) $$\hat{y}=f\left(x\right)=b_{1}x_{1}+b_{2}x_{2}+\ldots +b_{M}x_{M}=x^{T}b$$

where *x*_1_, *x*_2_ … *x*_*M*_ are the components of the vector of measurements ***x*** and the transpose indicates that it is normally expressed as a row vector, while the associated linear coefficients *b*_1_, *b*_2_ … *b*_*M*_, which weight the contributions of each of the *M X*-variables to *y*, are called regression coefficients and collected in the vector ***b***. Building a regression model means to find the optimal value of the parameters ***b***, i.e., the values which lead to the lowest error in the prediction of the responses. As a direct consequence of this consideration, it is obvious how it is mandatory to have a set of samples (the so-called *training set*) for which both the experimental data ***X*** and the responses ***y*** are available, in order to build a predictive model. Indeed, the information on the ***y*** is actively used to calculate the model parameters. When data from more than a single sample are available, the regression problem in Equations (2, 3) can be reformulated as:

(4) $$y=\hat{y}+e=Xb+e$$

where the vectors $\hat{y}$ and ***e*** collect the predictions and residuals for the different samples, respectively. Accordingly, the most straightforward way of calculating the model parameters in Equation (4) is by the ordinary least-squares approach, i.e., by looking at those values of ***b***, which minimize the sum of squares of the residuals ***e***:

(5) $$\underset{b}{\min}e^{T}e=\underset{b}{\min}\sum_{i=1}^{N}e_{i}^{2}$$

*e*_*i*_ being the residual for the i^th^ sample and *N* being the number of training observations. The corresponding methods is called multiple linear regression (MLR) and, under the conditions of Equation (5), the regression coefficients are calculated as:

(6) $$\begin{array}{c} b={\left(X^{{}^{T}}X\right)}^{-1}X^{{}^{T}}y \end{array}$$

Here it is worth to highlight that, if one wishes to use the same experimental matrix ***X*** to predict more than one response, i.e., if, for each sample, instead of a single scalar *y*_*i*_, there is a dependent vector

(7) $$y_{i}^{T}=\left[y_{i1}y_{i2}\cdots y_{iL}\right]$$

*L* being the number of responses, then each dependent variable should be regressed on the independent block by means of a set of regression coefficients. Assuming that the *L* responses measured on the training samples are collected in a matrix ***Y***, whose columns ***y***_*l*_ are the individual dependent variables,

(8) $$Y=\left[y_{1}\cdots y_{l}\cdots y_{L}\right]$$

the corresponding regression equations could be written as:

(9) $$\begin{array}{c} \begin{array}{c} y_{1}=Xb_{1}+e_{1}\\ \vdots \\ y_{l}=Xb_{l}+e_{l} \end{array}\\ \begin{array}{c} \vdots \\ y_{L}=Xb_{L}+e_{L} \end{array} \end{array}$$

which can be grouped into a single expression:

(10) Y=XB+E

where the residuals, i.e., the differences between the measured and predicted responses are collected in the matrix ***E***, and the regression coefficients vectors are gathered in a matrix ***B***, which can be estimated, analogously to Equation (6), as:

(11) $$\begin{array}{c} B=\left[b_{1}\cdots b_{l}\cdots b_{L}\right]={\left(X^{{}^{T}}X\right)}^{{}^{-1}}X^{T}Y. \end{array}$$

Equations (9–11) indicate that, as far as MLR is concerned, building a model to predict one response at a time or another model to predict multiple responses altogether would lead to the same results since, in the latter case, each dependent variable is anyway modeled as if it were alone. In either case, the solutions of the least-squares problem reported in Equations (6, 11) rely on the possibility of inverting the matrix (***X***^*T*^***X***), i.e., on the characteristics of the predictors. Indeed, in order for that matrix to be invertible, the number of samples should be higher than that of variables and the variables themselves should be as uncorrelated as possible. These conditions are rarely met by the techniques which are used to characterize pharmaceutical samples and, in particular, never met by spectroscopic methods. Due to these limitations, alternative approaches have been proposed in the literature to build regression models in cases where standard multiple linear regression is not applicable. In particular, since in order for the regression solution to exist, the predictor matrix should be made of few, uncorrelated variables, most of the alternative approaches proposed in the literature involve the projection of the ***X*** matrix onto a reduced space of orthogonal components and the use of the corresponding scores as regressors to predict the response(s). In this regard, one of the most widely used approaches is principal component regression (PCR) (Hotelling, 1957; Kendall, 1957; Massy, 1965; Jeffers, 1967; Jolliffe, 1982, 2002; Martens and Naes, 1991 Martens and Geladi, 2004) which, as the name suggests, involves a two-stage process where at first principal component analysis is used to compress the information in the ***X*** block onto a reduced set of relevant scores, as already described in Equation (1):

(12) T=XP

and then these scores constitute the predictor block to build a multiple linear regression:

(13) $$\hat{Y}=TC$$

***C*** being the matrix of regression coefficients for this model. By combining Equations (12, 13), it can be easily seen how PCR still describes a linear relationship between the responses ***Y*** and the original variables ***X***:

(14) $$\hat{Y}=TC=XPC=XB_{PCR}$$

mediated by a matrix of regression coefficients ***B***_*PCR*_(=***PC***), which is different from the one that would be estimated by Equation (11), since it is calculated by taking into account only the portion of the variability in the ***X*** block accounted for by the selected principal components. The use of principal component scores as predictors allows to solve the issues connected to the matrix (***X***^*T*^***X***) being usually ill-conditioned when dealing with spectroscopic techniques, but may be still suboptimal in terms of predictive accuracy.

Indeed, as described in Equations (12, 13), calculating a PCR model is a two-step procedure, which involves at first the calculation of PC scores and then the use of these scores to build a regression model to predict the response(s). However, these two steps have different objective functions, i.e., the criterion which is used to extract the scores from the ***X*** matrix is not the same which guides the calculation of the regression coefficients ***C*** in Equation (13). Stated in different words, the directions of maximum explained variance (especially when there are many uninformative sources of variability in the data) may not be relevant for the prediction of the ***Y***. To overcome this drawback, an alternative approach to component-based regression is represented by the Partial Least-Squares algorithm (Wold et al., 1983; Geladi and Kowalski, 1986; Martens and Naes, 1991) which, due to its being probably the most widely used calibration method in chemometrics, will be described in greater detail in the following subparagraph.

### Partial least squares (PLS) regression

Partial Least Squares (PLS) regression (Wold et al., 1983; Geladi and Kowalski, 1986; Martens and Naes, 1991) was proposed as an alternative method to calculate reliable regression models in the presence of ill-conditioned matrices. Analogously to PCR, it is based on the extraction of a set of scores ***T*** by projecting the ***X*** block on a subspace of latent variables, which are relevant for the calibration problem. However, unlike PCR, the need for the components not only to explain a significant portion of the ***X*** variance but also to be predictive for the response ***Y*** is explicitly taken into account for the definition of the scores. Indeed, in PLS, the latent variables (i.e., the directions onto which the data are projected) are defined in such a way to maximize the covariance between the corresponding scores and the response(s): maximizing the covariance allows to obtain scores which at the same time describe a relevant portion of the ***X*** variance and are correlated with the response(s). Due to these characteristics, and differently than what already described in the case of MLR (see Equation 11) and, by extension, PCR, in PLS two distinct algorithms have been proposed depending on whether only one or multiple responses should be predicted (the corresponding approaches are named PLS1 and PLS2, respectively). In the remainder of this section, both algorithms will be briefly described and commented.

When a single response has to be predicted, its values on the training samples are collected in a vector ***y***; accordingly, the PLS1 algorithm extracts scores from the ***X*** block having maximum covariance with the response. In particular, the first score ***t***_1_ is the projection of the data matrix ***X*** along the direction of maximum covariance ***r***_1_:

(15) $$\underset{{\;}_{r_{1}}}{\max}\;[Cov(t_{1},y)]=\underset{{\;}_{r_{1}}}{\max}\;(t_{1}^{T}y)$$

While the successive scores ***t***_2_ ⋯ ***t***_*F*_, which are all orthogonal, account in turn for the maximum residual covariance. Therefore, PLS1 calculates a set of orthogonal scores having maximum covariance with ***y***, according to:

(16) T=XR

***R*** being the weights defining the subspace onto which the matrix should be projected, and then uses these scores as regressors for the response:

(17) $$\hat{y}=Tq$$

***q*** being the coefficients for the regression. Similarly to what already shown in the case of PCR, Equations (16, 17) can be then combined in a single one to express the regression model as a function of the original variables, through the introduction of the regression vector ***b***_*PLS*1_ (=***Rq***):

(18) $$\hat{y}=Tq=\;XRq=Xb_{PLS1}.$$

In contrast, in the multi-response case (PLS2), it is assumed that also the matrix ***Y***, which collects the values of the dependent variables on the training samples, has a latent structure, i.e., it can be approximated by a component model:

(19) $$\hat{Y}=UQ^{T}$$

***U*** and ***Q*** being the ***Y*** scores and loadings, respectively. In particular, in order for the calibration model to be efficient, it is assumed that the ***X*** and the ***Y*** matrices share the same latent structure. This is accomplished by imposing that the component be relevant to describe the variance of the independent block and predictive for the responses. In mathematical terms, pairs of scores are simultaneously extracted from the ***X*** and the ***Y*** blocks so to have maximum covariance:

(20) $$\underset{{\;}_{r_{i},q_{i}}}{\max\;}[Cov(t_{i},u_{i})]=\underset{{\;}_{r_{i},q_{i}}}{\max\;}(t_{i}^{T}u_{i})$$

Where ***t***_*i*_ and ***u***_*i*_ are the ***X*** and the ***Y*** scores on the ith latent variable, respectively, ***q***_*i*_ is the ith column of the ***Y*** loading matrix ***Q*** while ***r***_*i*_ is the ith column of the ***X*** weight matrix ***R***, which has the same meaning as specified in Equation (16). Additionally, these scores are made to be collinear, through what is normally defined as the inner relation:

(21) $$u_{i}=t_{i}c_{i}\;\forall i$$

*c*_*i*_ being a proportionality constant (*inner regression coefficient*). When considering all the pairs of components, Equation (21) can be rewritten in a matrix form as:

(22) U=TC

where

(23) $$C=\left[\begin{array}{ccc} c_{1} & \cdots & 0\\ \vdots & \ddots & \vdots \\ 0 & \cdots & c_{F} \end{array}\right].$$

Also in this case, by combining all the equations defining the model, it is possible to express the predicted responses as a linear function of the original variables:

(24) $$\hat{Y}=UQ^{T}=TCQ^{T}=XRCQ^{T}=XB_{PLS2}$$

where the matrix of regression coefficients ***B***_*PLS*2_ is defined as ***RCQ***^*T*^.

Based on the above description, it is clear that, when more than one response has to be modeled, it is essential to decide whether it could be better to build an individual model for each dependent variable, or a single model to predict all the responses, as the results would not be the same. In particular, it is advisable to use the PLS2 approach only when one could reasonably assume that there are systematic relationships between the dependent variables.

On the other hand, independently on what model one decides to use, once the values of the regression coefficients (here generally indicated as ***B***) have been estimated based on the training samples, they can be used to predict the responses for any new set of measurements (***X***_*new*_):

(25) $${\hat{Y}}_{new}=X_{new}B.$$

Here, it should be stressed that, in order for the calibrations built by PLS (but the same concept holds for PCR) to be accurate and reliable, a key parameter is the choice of an appropriate number of latent variables to describe the data. Indeed, while selecting a low number of components one can incur in the risk of not explaining all the relevant variance (*underfitting*), including too many of them (so that not only the systematic information is captured, but also the noise), can lead to *overfitting*, i.e., to a model which is very good in predicting the samples it has been calculated on, but performs poorly on new observations. To reduce this risk, a proper validation strategy is needed (see section Validation) and, in particular, the optimal number of latent variables is selected as the one leading to the minimum error during one of the validation stages (usually, cross-validation).

### Selected application of regression methods to pharmaceutical problems

Regression methods in general, and especially PLS, are often combined with spectroscopy in order to develop rapid and (sometimes) non-destructive methodologies for the quantification of active ingredients in formulations. For instance, Bautista et al. (1996) quantified three analytes of interest (caffeine, acetylsalicylic acid and acetaminophen) in their synthetic ternary mixtures and different formulations by UV-Vis spectroscopy assisted by a PLS calibration model. Mazurek et al. proposed two approaches based on coupling FT-Raman spectroscopy with PLS and PCR calibration for estimation of captopril and prednisolone in tablets (Mazurek and Szostak, 2006a) and diclofenac sodium and aminophylline in injection solutions (Mazurek and Szostak, 2006b). The authors compared results obtained from calibration models built by using unnormalised spectra with the values found when an internal standard was added to each sample and the spectra were normalized by its selected band intensity at maximum or integrated. Another study on injection samples was proposed by Xie et al. (2010), using NIR spectroscopy combined with PLS and PCR to quantify pefloxacin mesylate (an antibacterial agent) in liquid formulations. PLS regression was also coupled to MIR (Marini et al., 2009) and NIR spectroscopy (Rigoni et al., 2014) to quantify the enantiometric excess of different APIs in the solid phase, also in the presence of excipients, based on the consideration that, in the solid phase, the spectrum of the racemic mixture could be different from that of either pure enantiomer. Specifically, it was possible to accurately quantify the enantiomeric excess of S-(+)-mandelic acid and S-(+)-ketoprofen by MIR spectroscopy coupled by PLS on the whole spectrum and after variable selection by sequential application of backward interval PLS and genetic algorithms (biPLS-GA) (Marini et al., 2009), while NIR was used to quantify the enantiomeric excess of R-(–)-epinephrine and S-(+)-ibuprofen (Rigoni et al., 2014). In the latter case, it was also shown that, when using the validated model to quantify the enantiomeric excess of API in the finished products, the influence of excipients and dosage forms (intact tablets or powders) has a relevant impact on the final predictive accuracy.

## Classification

As already introduced in the previous section, in chemometric applications, in general, and in the context of pharmaceutical analysis, in particular, one is often interested in using the experimentally collected data (e.g., spectroscopic profiles) to predict qualitative or quantitative properties of the samples. While the regression methods for the prediction of quantitative responses have been already presented and discussed in section Regression, the main chemometric approaches for the prediction of qualitative properties of the individuals under investigation are outlined herein. These approaches are generally referred to as classification methods, since any discrete level that the qualitative variable can assume may also be defined as a class (or category) (Bevilacqua et al., 2013). For instance, if one were interested in the possibility of recognizing which of three specific sites a raw material was supplied from, it is clear that the response to be predicted could only take three possible values, namely “Site A,” “Site B,” and “Site C”; each of these three values would correspond to a particular class. A class can be then considered as an ensemble of individuals (samples) sharing similar characteristics. In this example, samples from the first class would all be characterized by having been manufactured from a raw material produced in Site A, and similar considerations could be made for the specimens in the second and third classes, corresponding to Site B and Site C, respectively. As it could already be clear from the example, there are many ambits of application for classification methods in pharmaceutical and biomedical analysis, some of which will be further illustrated in section Selected Applications of Classification Approaches for Pharmaceutical Analysis, after a brief theoretical introduction to the topic as well as the chemometric methods most frequently used in this context (especially, in combination with spectroscopic techniques).

**Figure 4:**
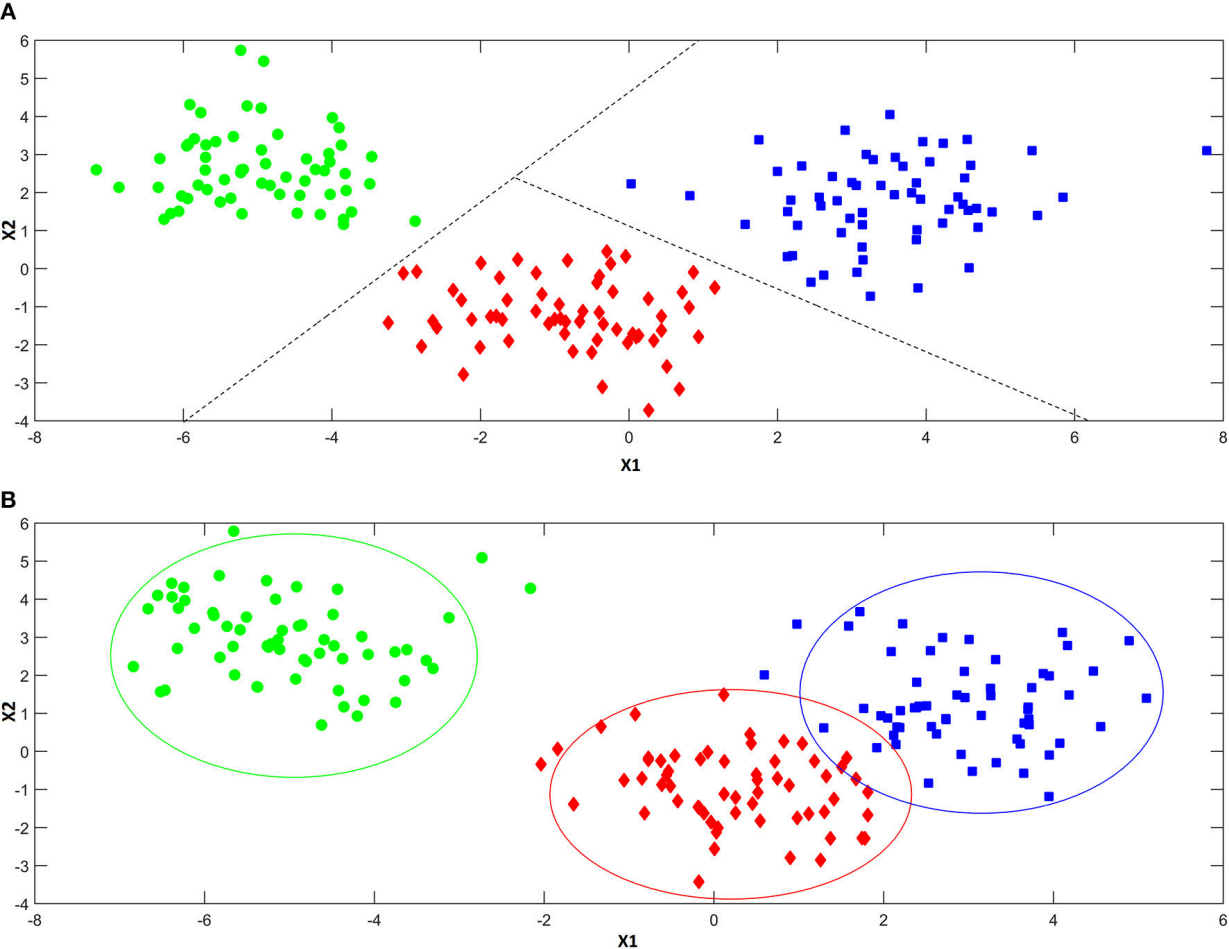
Illustration of the difference between discriminant **(A)** and modeling **(B)** classification techniques. Discriminant classification techniques **(A)** divide the available hyperspace into as many regions as the number of the investigated categories (three, in the present example), so that whenever a sample falls in a particular region of space, it is always assigned to the associated class. Modeling techniques **(B)** build a separate model for each one of the categories of interest, so that there can be regions of spaces where more than a class is mapped and others where there is no class at all.

As anticipated above, classification approaches aim at relating the experimental data collected on a sample to a discrete value of a property one wishes to predict. This same problem can be also expressed in geometrical terms by considering that each experimental profile (e.g., spectrum) can be seen as point in the multivariate space described by the measured variables. Accordingly, a classification problem can be formulated as the identification of regions in this multivariate space, which can be associated to a particular category, so that if a point falls in one of these regions, it is predicted as being part of the corresponding class. In this respect, classification approaches can be divided into two main sub-groups: discriminant and class-modeling methods. In this framework, a fundamental distinction can be made between discriminant and class-modeling tools, which constitute the two main approaches to perform classification in chemometrics (Albano et al., 1978). In detail, discriminant classification methods focus on identifying boundaries in the multivariate space, which separate the region(s) corresponding to a particular category from those corresponding to another one. This means they need representative samples from all the categories of interest in order to build the classification model, which will be then able to predict any new sample as belonging only to one of the classes spanned by the training set. In a problem involving three classes, a discriminant classification method will look for those boundaries in the multivariate space identifying the regions associated to the three categories in such a way as to minimize the classification error (i.e., the percentage of samples wrongly assigned). An example is reported in Figure 4A. On the other hand, class-modeling techniques look at the similarities among individuals belonging to the same category, and aim at defining a (usually bound) subspace where samples from the class under investigation can be found with a certain probability; in this sense, they resemble outlier tests, and indeed they borrow most of the machinery from the latter. Operationally, each category is modeled independently on the others and the outcome is the definition of a class boundary which should enclose the category sub-space:, i.e., individuals falling within that space are likely to belong to the class (are “accepted” by the class model), whereas samples falling outside are deemed as outliers and rejected. It is then evident that one of the main advantages of class modeling approaches is that they allow building a classification model also in the asymmetric case, where there is only a category of interest and the alternative one is represented by all the other individuals not falling under the definition of that particular class. In this case, since the alternative category is ill-defined, heterogeneous, and very likely to be underrepresented in the training set, any discriminant model would result suboptimal, as its predictions would strongly depend on the (usually not enough) samples available for that class. On the other hand, modeling techniques define the category space only on the basis of data collected for the class of interest, so those problems can be overcome.

When the specific problem requires to investigate more than one class, each category is modeled independently on the others and, accordingly, the corresponding sub-spaces may overlap (see Figure 4B). As a consequence, classification outcomes are more versatile than with discriminant methods: a sample can be accepted by a single category model (and therefore be assigned to that class), by more than one (falling in the area where different class spaces overlap and, hence, resulting “confused”) or it could fall outside any class-region and therefore be rejected by all the categories involved in the model.

### Discriminant methods

As mentioned above, predictions made by the application of discriminant methods are univocal; namely, each sample is uniquely assigned to one and only one of the classes represented in the training set. This is accomplished by defining decision surfaces, which delimit the boundaries among the regions of space associated to the different categories. Depending on the model complexity, such boundaries can be linear (hyperplanes) or assume more complex (non-linear) shapes. When possible, linear discriminant models are preferred as they have less parameters to tune, require a lower number of training samples and are in general more robust against overfitting. Based on these considerations, the first-ever and still one of the most commonly used discriminant techniques is Linear Discriminant Analysis (LDA), originally proposed by Fisher (1936). It relies on the assumption that the samples of each class are normally distributed around their respective centroids with the same variance/covariance matrix (i.e., the same within-category scatter). Under these assumptions, it is possible to calculate the probability that each sample belongs to a particular class *g p*( *g*|***x***), as:

(26) $$p\left(\;g|x\right)=\frac{\pi_{g}}{C}e^{-\frac{1}{2}{\left(x-{\bar{x}}_{g}\right)}^{T}S^{-1}\left(x-{\bar{x}}_{g}\right)}$$

where ${\bar{x}}_{g}\;$is the centroid of class *g*, ***S*** the overall within-class variance/covariance matrix, π_*g*_ the *prior* probability (i.e., the probability of observing a sample from that category before carrying out any measurement), *C* is a normalization constant and the argument of the exponential ${\left(x-{\bar{x}}_{g}\right)}^{T}S^{-1}\left(x-{\bar{x}}_{g}\right)$ is defined as the squared Mahalanobis distance of the individual to the center of the category. Classification is then accomplished by assigning the sample to the category, to which it has the highest probability of belonging.

LDA is a well-established technique, which works well also on data for which the normality assumption is not fulfilled but, unfortunately, it can rarely be used on spectroscopic data for the same reasons MLR cannot be utilized for regression (see section Regression): calculation of matrix ***S***^−1^ requires the experimental data matrix to be well-conditioned, which is not the case, when dealing with a high number of correlated variables measured on a limited number of samples. To overcome these limitations, LDA can be applied on the scores of bilinear models used to compress the data (e.g., on principal components), but the most commonly used approach involves a suitable modification of the PLS algorithm which makes it able to deal with classification issues; the resulting method is called partial least squares discriminant analysis (PLS-DA) (Sjöström et al., 1986; Ståle and Wold, 1987; Barker and Rayens, 2003), and it will be briefly described in the following paragraph.

#### Partial least squares discriminant analysis (PLS-DA)

In order for the PLS algorithm to deal with discriminant classification problems, the information about class belonging has to be encoded in a response variable ***Y***, which can be then regressed onto the experimental matrix ***X*** to provide the predictive model (Sjöström et al., 1986). This is accomplished by defining ***Y*** as a “dummy” binary matrix, having as many rows as the number of samples (*N*) and as many columns as the number of classes (*G*). Each row in ***Y*** is a vector encoding the information about class belonging of the corresponding sample, whereas each column is associated to a particular class (the first column to class 1, the second to class 2 and so on up to the *G*^th^). As such, the row vector corresponding to a particular sample will contain all zeros except for the column associated to the class it belongs to, where there will be a one. For instance, in the case of a problem involving three categories, a sample belonging to Class 2 will be represented by the vector ***y***_*i*_ = [0 1 0]. A PLS regression model is then calculated between the experimental data matrix ***X*** and the dummy ***Y*** [as described in section Partial Least Squares (PLS) Regression], and the matrix of regression coefficients obtained is used to predict the value of the responses on new samples, ${\hat{Y}}_{new}$. Since the dependent variable is associated to the categorical information, classification of the samples is based on the predicted responses ${\hat{Y}}_{new}$ which, however, are not binary but real-valued. As a consequence, different approaches have been proposed in the literature to define how to classify samples in PLS-DA based on the values of ${\hat{Y}}_{new}$. The naivest approach (see e.g., Alsberg et al., 1998) is to assign each sample to the category corresponding to the highest value of the predicted response vector. For instance, if the following predictions were obtained for a particular sample: ${\hat{y}}_{new,k}=\left[0.1\;-0.4\;0.8\right]$, it would be assigned to Class 3. On the other hand, other strategies have been also suggested, like the application of LDA on ${\hat{Y}}_{new}$ or on the PLS scores (Nocairi et al., 2004; Indahl et al., 2007), or the use of thresholds based on probability theory (Pérez et al., 2009).

### Class-modeling methods

As already stated, class-modeling methods aim at identifying a closed (bound) sub-space, where it is likely to find samples from a particular category, irrespective of whether other classes should also be considered or not. They try to capture the features, which make individuals from the same category similar to one another. Operationally, they define the class space by identifying the “normal” variability which can be expected among samples belonging to that category and, accordingly, introducing a “distance-to-the-model” criterion which accounts for the degree of outlyingness of any new sample. Among the different class-modeling techniques proposed in the literature, soft independent modeling of class analogies (SIMCA) is by far the most commonly used, especially for spectroscopic data, due to its ability of dealing with ill-conditioned experimental data matrices and, therefore, it will be briefly described below (for more details, the reader is referred to Wold, 1976; Wold and Sjöström, 1977, 1987).

#### Soft independent modeling of class analogies (SIMCA)

The main idea behind SIMCA is that the systematic variability characterizing the samples for a particular category can be captured and accurately accounted for by a PCA model of appropriate dimensionality. This model is built by using only the samples from the investigated category:

(27) $$X_{g}=T_{g}P_{g}^{T}+E_{g}$$

where the symbols have the same meaning as in Equation (2), and the subscript indicates that the model is calculated by using only the training data from class *g*. The use of PCA to define the similarities among the samples belonging to the category of interest provides also the machinery to assess whether any new sample is likely to come from that class or not through the definition of two statistics normally used for outlier detection, namely *T*^2^ and *Q*. As already introduced in section Principal Component Analysis, the former is the squared Mahalanobis distance of a sample to the center of the scores space, indicating how far the individual is from the distribution of the “normal” samples in the space spanned by the significant PCs (Hotelling, 1931), while the latter is the (Euclidean) distance of the sample to its projection onto the PC space, describing how well that individual is fitted by the PCA model (Jackson and Muldholkar, 1979). In the context of SIMCA, once the PCA model of the g^th^ category is calculated according to Equation (27), any specimen to be predicted is projected onto that model and its values of *T*^2^ and *Q* are used to calculate an overall distance to the model *d*_*i, g*_ (Yue and Qin, 2001), which constitutes the basis for class acceptance or rejection:

(28) $$d_{i,g}=\sqrt{{\left(T_{i,g}^{2}\right)}^{2}+{\left(Q_{i,g}\right)}^{2}}$$

where the subscript indicates that the i^th^ sample is tested against the model of the g^th^ category. Accordingly, the boundary of the class space is identified by setting a proper threshold to the distance, so that if a sample has a distance to the model lower than the threshold it is accepted by the category and, otherwise, it is rejected.

### Selected applications of classification approaches for pharmaceutical analysis

As mentioned before, classification approaches are widely applied in quality controls of pharmaceuticals, in particular to detect counterfeit drugs, as, for instance, it is reported in da Silva Fernandes et al. (2012), where NIR and fluorescence spectroscopy were combined with different classification methods to distinguish among pure and adulterated tablets. In Storme-Paris et al. (2010), a non-destructive approach is proposed to distinguish genuine tablets from counterfeit or recalled (from the market) medicines. In order to achieve this, NIR spectra (directly collected on the tables) are analyzed by SIMCA. Results obtained suggest the validity of this approach; in fact, it allowed highlighting small differences among drugs (e.g., different coating), and it provided an excellent differentiation among genuine and counterfeits products. For the same purpose, namely counterfeit drug detection, NIR spectra were also widely combined with PLS-DA. Only to mention one, de Peinder et al. (2008) demonstrated the validity of this approach to spot counterfeits of a specific cholesterol-lowering medicine. Despite the fact that the authors highlighted the storage conditions sensibly affecting NIR spectra (because of humidity), the PLS-DA model still proved to be robust and provided excellent predictions.

## Validation

Chemometrics relies mainly on the use of empirical models which, given the experimental measurements, should summarize the information of the data, reasonably approximate the system under study, and allow predictions of one or more properties of interest. Bearing this in mind, given the “soft” (i.e., empirical) nature of the models employed, there are many models one could in principle calculate on the same data and their performances could be influenced by different factors (number of samples and their representativeness, the method itself, the algorithm, and so on) (Brereton et al., 2018). Thus, selecting which model is the most appropriate for the data under investigation and verifying how reliable it is, is of fundamental importance and the chemometric strategies for doing so are collectively referred to as validation (Harshman, 1984; Westad and Marini, 2015). To evaluate the quality of the investigated models, the validation process requires the definition of suitable diagnostics, which could be based on model parameters but more often rely on the calculation of some sort of residuals (i.e., error criteria). In this context, in order to avoid overoptimism or, in general, to obtain estimates which are as unbiased as possible, it is fundamental that the residuals which are used for validation are not generated by the application of the model to the data it has been built on, since in almost all cases, they cannot be considered as representative of the outcomes one would obtain on completely new data. For such reason, a correct validation strategy should involve the estimation of the model error on a dataset different than the one used for calculating the model parameters. This is normally accomplished through the use of an external test set or cross-validation.

The use of a second, completely independent, set of data for evaluating the performances and, consequently, calculating the residuals (test set validation) is the strategy which best mimics how the model will be routinely used, and it is therefore the one to be preferred, whenever possible. On the other hand, cross-validation is based on the repeated resampling of the dataset, into a training and a test sub-sets, so that at each iteration only a part of the original samples is used for model building while the remaining individuals are left out for validation. This procedure is normally repeated up to the moment when each sample has been left out at least once or, anyway, for a prespecified number of iterations. Cross-validation is particularly suited when the number of available samples is small and there is no possibility of building an external test set, but the resulting estimates can be still biased as the calibration and validation sets are never completely independent on one another. In general, it is rather used for model selection (e.g., estimating the optimal number of components) than for the final validation stage.

## Other selected applications

In addition to some specific applications described above, in this paragraph additional examples will be presented to further emphasize the usefulness of chemometrics-based spectroscopy for pharmaceutical analysis.

Morris and Forbes (2001) coupled NIR spectroscopy with multivariate calibration for quantifying narasin chloroform-extracted from granulated samples. In another study, Forbes et al. (2001) proposed a transmission NIR spectroscopy method using multivariate regression for the quantification of potency and lipids in monensin fermentation broth.

Ghasemi and Niazi (2007) developed a spectrophotometric method for the direct quantitative determination of captopril in pharmaceutical preparation and biological fluids (human plasma and urine) samples. Since the spectra were recorded at various pHs (from 2.0 to 12.8), different models were tested, including the possibility of a preliminary spectral deconvolution using multi-way approaches. In particular, the use of PLS on the spectra at pH 2.0 allowed to build a calibration curve which resulted in a very good accuracy. Li et al. (2014) used Raman spectroscopy to identify anisodamine counterfeit tablets with 100% predictive accuracy and, at the same time, NIR spectroscopy to discriminate genuine anisodamine tablets from 5 different manufacturing plants. In the latter case, PLS-DA models were found to have 100% recognition and rejection rates. Willett and Rodriguez (2018) implemented a rapid Raman assay for on-site analysis of stockpiled drugs in aqueous solution, which was tested on Tamiflu (oseltamivir phosphate) by using three different portable and handheld Raman instruments. PLS regression models yielded an average error with respect to the reference HPLC values, which was lower than 0.3%. Other examples of application can be found in Forina et al. (1998), Komsta (2012), Hoang et al. (2013), and Lohumi et al. (2017).

## Conclusions

Chemometrics provide a wealth of techniques for both the exploratory analysis of multivariate data as well as building reliable calibration and classification strategies to predict quantitative and qualitative responses based on the experimental profiles collected on the samples. Coupled to spectroscopic characterization, it represents an indispensable and highly versatile tool for pharmaceutical analysis at all levels.

## Author contributions

AB and FM jointly conceived and designed the paper, and wrote the manuscript. All authors agreed on the content of the paper and approved its submission.

### Conflict of interest statement

The authors declare that the research was conducted in the absence of any commercial or financial relationships that could be construed as a potential conflict of interest.
